# Diet with a High Proportion of Rice Alters Profiles and Potential Function of Digesta-Associated Microbiota in the Ileum of Goats

**DOI:** 10.3390/ani10081261

**Published:** 2020-07-24

**Authors:** Kaijun Wang, Ao Ren, Mengli Zheng, Jinzhen Jiao, Qiongxian Yan, Chuanshe Zhou, Zhiliang Tan

**Affiliations:** 1 CAS Key Laboratory of Agro-Ecological Processes in Subtropical Region, National Engineering Laboratory for Pollution Control and Waste Utilization in Livestock and Poultry Production, Hunan Provincial Key Laboratory of Animal Nutrition & Physiology and Metabolism, Institute of Subtropical Agriculture, The Chinese Academy of Sciences, Changsha 410125, China; kj-wang@foxmail.com (K.W.); 18711326324@163.com (A.R.); 18274877387@163.com (M.Z.); jjz@isa.ac.cn (J.J.); zcs@isa.ac.cn (C.Z.); zltan@isa.ac.cn (Z.T.); 2 Hunan Co-Innovation Center of Animal Production Safety, CICAPS, Changsha 410128, China

**Keywords:** bacterial community, high-concentrate diet, goat, intestine, metabolites

## Abstract

**Simple Summary:**

Our study provided a detailed picture of the ileal microbiota related to a high-concentrate diet and showed that it could reduce the bacterial richness and diversity in the ileum of goats. A high-concentrate diet may inhibit the growth of intestinal bacterial communities. Ileal concentrations of total volatile fatty acids increased and pH decreased with a high-concentrate diet, which could increase the risk of poor health in growing goats. Such an understanding will be essential to predict variations in the gastrointestinal microbiota to enhance the productivity and welfare of ruminants through nutritional strategies, such as diet intervention.

**Abstract:**

Effects of a high proportion of concentrate in the diet on the ileal microbiota and metabolites in small ruminants are rarely reported. This study was designed to investigate the ileal microbiota and its relationship with host metabolic function in goats and aimed to elucidate the mechanisms involving in the ileal adaptation to a diet containing a high proportion of rice. Sixteen goats were equally divided into two groups and fed a diet with a normal concentrate proportion (NC, 55% concentrate) or a high-concentrate diet (HC, 90% concentrate). Results showed that the HC diet decreased bacterial diversity and elevated the abundance of five genera (*Clostridium*_*sensu*_*stricto*_*1*, *Eubacterium*_*nodatum*_group, *Ruminococcus*_*gauvreauii*_group, *Eubacterium*_*coprostanoligenes*_group and *Ruminococcus* 1), but reduced the number of Anaerotruncus. Microbial functional potentials indicated that the HC diet activated the pathways related to metabolism of carbohydrate, glycan, lipid and vitamins, but inhibited the pathways associated with cell motility and signal transduction. The activities of amylase and alkaline phosphatase were greater (*p* < 0.05) in the intestinal digesta of the HC-fed goats. However, there were no differences in the villus height, crypt depth and the ratio of villus height to crypt depth in the ileum between the two groups. These results indicate that the HC diet alters the bacterial community and pathways related to the metabolism of dietary nutrients and cell motility and signal transduction of bacteria in the ileum of goats.

## 1. Introduction

In modern ruminant production systems, to achieve maximum performance, the inclusion of plentiful amounts of concentrate in the diet is commonly practiced in the intensive feedlot management system of dairy cows and goats [1,2]. Relatively high fiber feeding and stable microbial community are the indispensable conditions for the health of ruminants [3]. However, at present, the high-concentrate (HC) diet is widely adopted to provide adequate protein and energy supply for meeting higher performance needs at the finishing stage of ruminants [4]. Appropriate increase of dietary concentrate level can improve the performance of ruminants [5], whereas high inclusion rates of concentrates affects the conditions of microorganisms inhabiting the gastro-intestines, including the acidity, osmolality, and the contents of fermentable substrates [6,7]. Dietary starch content can affect the rumen and hindgut bacteria when raising dietary concentrate proportion, but these effects vary greatly among ruminant species [8,9].

The animal digestive tract is colonized by a dense, dynamic and highly complex community of microorganisms composed mainly of bacteria, whose total number exceeds 10^14^ cells, with many unique strains [10]. Intestinal microflora play a key role in nutrient metabolism and development of the immune system [11,12]. Among the elements affecting bacteria in the intestine, a major factor is the ability to utilize the available nutrients to achieve high growth rates to avoid washout and appease a reaction-ready immune system [13]. The large amount of carbohydrates promotes the growth of bacteria in the small intestine [14]. In contrast, in the hindgut, plenty of nutrients for bacteria are derived from undigested carbohydrates and resistant starch as well as undigested protein in the diets. There these nutrients undergo microbial fermentation, resulting in the production of metabolites, such as VFA (volatile fatty acid) [15]. Many previous studies have revealed that an increasing fermentation in the rumen and hindgut typically occurs concurrently with HC feeding and increments of VFA and LPS (lipopolysaccharide), as well as reduced pH in the hindgut of ruminants, causing an unhealthy gastrointestinal environment [16,17]. Such studies were necessary in order to enhance the understanding of the relationship between HC diet and the intestinal bacteria of goats, as this would lay a foundation for the development of strategies to prevent this disorder.

The development and application of Illumina MiSeq sequencing methods may help to explain the underlying mechanism of comprehensive variation of intestinal bacteria caused by feeding HC diet. Our previous studies have noted that feeding a HC diet causes subacute ruminal acidosis [18] and alters total VFA production in the ileum [19], indicating that a HC diet challenge increases the risk of poor gastrointestinal health. Therefore, in the present study, we hypothesized that a HC diet might cause changes in the ileal metabolic activities and bacterial community, and further induce ileal epithelial injury in goats. The objective of this study was to investigate the changes in the ileal microbiota, metabolites, biochemical parameters and histomorphology during HC feeding. Additionally, the relationship among alterations in the ileal bacterial composition and metabolites and biochemical indices in the intestinal digesta were also evaluated. The reason for choosing rice grain as the concentrate supplements was that rice is planted on a large scale in the south of China.

## 2. Materials and Methods

The Laboratory Animal Welfare and Animal Experimental Ethical Inspection Committee at the Institute of Subtropical Agriculture, Chinese Academy of Sciences reviewed and approved all protocols used in this study. The ethical code is No. ISA000185. The animal trials were conducted at the experimental farm of the Institute of Subtropical Agriculture (Changsha, China).

### 2.1. Animals, Diets and Management

Sixteen Liuyang Black goats (six months old, a local breed in the south of China), with an average BW (body weight)of 15.3 ± 1.67 kg were randomly divided into two groups and fed either a normal control (NC, *n* = 8, the ratio of concentrate to forage was 55:45) diet or an HC (*n* = 8, the ratio of concentrate to forage was 90:10) diet, respectively. The ingredients and nutrient levels of the diets are shown in Table 1. Experiment duration consisted of seven days for diet adaptation and 28 days for sampling. Diet roughage was fed before concentrate and equal amounts were offered at approximately 08:00 and 18:00 h, respectively. All goats were fed in separate cages. Goats had free access to water, and the data of dry matter intake (DMI) and degradability was published in our previous study [18]. Finally, there were no significant differences in DMI and final body weight of goats, but the apparent digestibilities of NDF (neutral detergent fiber) and ADF (acid detergent fiber)were reduced by HC diet [18].

### 2.2. Measurements and Analytical Methods

Six goats in each group were slaughtered on day 35 after fasting for 12 h. Ileal contents were collected and then stored at −80 °C for microbial and metabolic analyses, and determination of fermentation products. Samples of ileal tissues from each goat were immediately fixed in 4% polyformaldehyde for intestinal morphological determination.

The total bacterial DNA of ileal contents was extracted according to the protocol [21] of the DNA Stool Mini Kit (Qiagen, Hilden, Germany). The bacterial universal V3-V4 region of the 16S rRNA gene was amplified according to PCR barcoded primers 338F (5′-ACTCCTACGGGAGGCAG-CAG-3′) and 806R (5′-GGACTACHVGGGTWTCTAAT-3′). PCR was performed in a total of 20 μL volumes, containing 1 × FastPfu Buffer, 250 μM dNTP, 0.1 μM each of the primer, 1 U FastPfu Polymerase (Beijing TransGen Biotech, Beijing, China) and 10 ng template DNA. PCR was performed at 95 °C for 2 min, followed by 30 cycles of 95 °C for 30 s, annealing at 55 °C for 30 s, 72 °C for 30 s, and a final extension at 72 °C for 5 min.

PCR products were identified using 2% agarose gel electrophoresis, purified with AxyPrep DNA Purification kit (Axygen Biosciences, Union City, CA, USA). The PCR products were visualized on agarose gels and quantitatively determined using QuantiFluor-ST Fluoremeter (Promega, Wisconsin, DC, USA) and PicoGreen dsDNA Quantitation Reagent (Invitrogen, Carlsbad, CA, USA). Purified amplicons were pooled in equimolar and paired-end sequenced (2 × 300) on an Illumina MiSeq platform (Allwegene, Beijing, China) according to the standard protocols.

### 2.3. Bacterial Data Processing and Function Predication

The raw reads files (fastq format) were de-multiplexed and quality-filtered using QIIME (version 1.17, Rob Knight et al., Department of Chemistry and Biochemistry, University of Colorado, Boulder, CO, USA. http://qiime.org/index.html) with the following criteria: (i) The 300 bp reads were truncated at any site that obtained an average quality score of <20 over a 10 bp sliding window, and the truncated reads shorter than 50 bp were discarded; (ii) exact barcode matching, two nucleotide mismatch in primer matching, and reads containing ambiguous characters were removed; (iii) only overlapping sequences longer than 10 bp were assembled according to their overlapped sequence. Reads that could not be assembled were discarded. Operational taxonomic units (OTUs) with 97% similarity cutoff were clustered using UPARSE (version 7.1, Edgar Robert C, Independent Investigator, Tiburon, CA, USA. http://drive5.com/uparse/), and chimeric sequences were identified and removed using UCHIME (a new program that detects chimeric sequences with two or more segments). The rarefaction analysis based on Mothur v.1.21.1 was conducted to reveal the diversity indices, including the Chao, Shannon, and coverage indices. The hierarchical clustering analysis was performed using the Primer 6 software (Primer-E Ltd., Plymouth, UK). Principal component analysis (PCA) was performed with Canoco 4.5. Venn diagrams were implemented by Venn Diagram, and community figures were generated using R tools according to the data from document “tax. phylum.xls, tax.family.xls, tax. genus.xls”. Phylogenetic investigation of communities by reconstruction of unobserved states (PICRUSt) was used as a bioinformatics tool to predict the functional potentials of metagenomes using 16S rRNA gene data [22]. The OTU table was imported into PICRUSt for functional gene predication by referencing the Kyoto Encyclopedia of Genes and Genomes (KEGG) database. Those pathways associated with human diseases and drug development were filtered out because they do not reflect microbial functions.

### 2.4. Metabolites Measured in the Ileal Digesta and Ileal Morphology

The ileal digesta was collected and kept in liquid nitrogen for further analysis of the metabolites. All the samples of digesta were homogenized thoroughly. The pH value and VFA content were presented in Table S1 (Supporting Information) with the data analyzed from our previous work [19]. The ileal metabolites including LACT (lactic acid), LDH (lactate dehydrogenase), ALT (alanine aminotransferase), AST (aspartate aminotransferase), ALP (alkaline phosphatase), and AMY (amylase) were detected with the commercial assay kits (Roche Diagnostics (Shanghai) Ltd., Shanghai, China; The intra- and inter-assay variation are shown in Table 2) and determined using an Automatic Biochemistry analyzer (Cobas c 311, Roche), and strictly following the manufacturer’s instructions, respectively. LPS in the ileum was detected using corresponding Enzyme-linked immunosorbent assay (ELISA) kit (Jiangsu Yutong Biological Technology Co., Ltd. Yancheng, China) with a minimum detection limit of 0.1 EU/mL. Pretreated supernatant was diluted until their LPS concentrations were in the range of 0.1 to 1 EU/mL relative to the reference endotoxin. The tissues were dehydrated and embedded following standard procedures. Formalin-fixed ileal samples were embedded in paraffin; cross sections of the segments were cut approximately 5 mm thick using a microtome and stained with hematoxylin and eosin. Three slides per goat, two images per slide, and a total of 36 replicates per group were harvested. In the present study, we used the pre-defined method which was reported by Wang et al. [23] to define the lesion. In each section, villus height and crypt depth were measured using a light microscope with a computer-assisted morphometric system. Villus height was defined as the distance from the villus tip to the crypt mouth, and crypt depth from the crypt mouth to the base.

### 2.5. Statistical Analyses

All statistical analyses were conducted with SPSS 19.0 (SPSS Inc., Chicago, IL, USA, 2009). Firstly, statistical analyses of data were evaluated through the Shapiro–Wilk test to check whether the distribution of the variables exhibited a normal distribution. Then, the variables that showed a normal distribution and a non-normal distribution were analyzed by the independent sample t test and the Kruskal–Wallis test, respectively. Statistical significance was set at *p* < 0.05 and tendencies at 0.05 ≤ *p* ≤ 0.10. Correlations between ileal parameters and microbiota were assessed by Pearson’s correlation test using GraphPad Prism version 6.00 (GraphPad Software, San Diego, CA, USA). Significance was declared at *p* < 0.05 and tendencies at 0.05 ≤ *p* ≤ 0.10.

## 3. Results

### 3.1. Ileal Bacterial Diversity and Similarity

**Figure 1:**
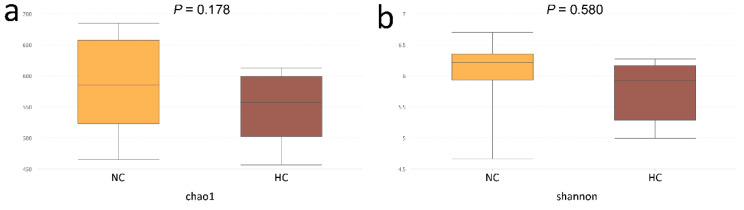
Alpha-diversity of intestinal bacterial community of goats fed normal-concentrate diet (NC) and high-concentrate diet (HC). (**a**) The bacterial richness in the ileum estimated by the Chao 1 value. (**b**) The bacterial diversity in the ileum estimated by Shannon index.

**Figure 2:**
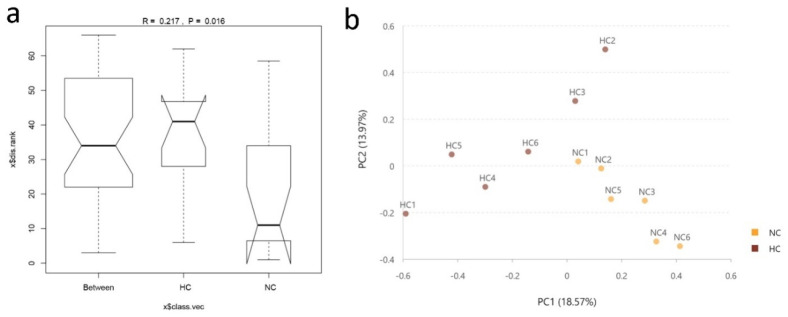
Similarity of intestinal bacterial community of goats fed NC and HC diets. (**a**) Analysis of similarities (Anosim) in the ileal bacterial community. (**b**) Principal component analysis (PCA) of ileal digestal bacterial community.

In total, after the size filtering, quality control and chimera removal, 391,601 valid sequences were obtained, with an average of 32,633 ± 6370 sequences per ileal sample. The overall OTU numbers classified at the distance level of 0.03 (97% similarity) were 1108 detected in the ileal samples, 998 in the NC group and 688 in the HC group, and 578 were shared by both. The bacterial community of the NC group had higher Chao estimate and Shannon index than the HC group (Figure 1a,b). When the ratio of dietary concentrate increased from 55% to 90%, Chao estimate of bacterial community decreased in the ileum of HC group. Meanwhile, the Shannon index of the bacterial community in the ileum was lower than the NC group. Bacterial communities of the ileal digesta (analysis of similarities (Anosim): *R* = 0.217, *p* = 0.016) were significantly affected by diet (Figure 2a). The NC group had a larger number of unique OTUs than the HC group. In addition, OTU community comparisons by principal component analysis (PCA) showed that samples from ileum in the NC group were separated from of those in the HC group (Figure 2b).

### 3.2. Intestinal Bacterial Community Structure

As shown in Table 3, Firmicutes, Tenericutes, Actinobacteria, Verrucomicrobia and Saccharibacteria were dominant phyla in the ileum of goats, accounting for more than 90% of the ileal total bacterial community. Some bacterial communities of phyla proportion varied with the increasing concentration of dietary concentrate. when concentrate concentration increased from 55% to 90%, the abundance of Chloroflexi (*p* = 0.058), Cyanobacteria (*p* = 0.090) and Euryarchaeota (*p* = 0.055) in the ileal digesta tended to be lower than in the NC group.

At family level, Firmicutes of the ileal bacteria community were mainly composed of Ruminococcaceae, Christensenellaceae, Lachnospiraceae, Peptostreptococcaceae and Family_XIII. Tenericutes were consisted of Mycoplasmataceae, and Actinobacteria were consisted of Coriobacteriaceae. As showed in Table S2, the proportion of Anaerolineaceae decreased dramatically from 1.08% to 0.02% after HC-fed (*p* = 0.058). No significant differences were observed in the proportions of the three dominant families between the NC and HC groups (*p* > 0.05).

Downward to genus levels, Christensenellaceae_R-7_group, *Peptoclostridium*, *Romboutsia*, Ruminococcaceae_NK4A214_group, Ruminococcaceae_UCG-014 and Saccharofermentans were the predominant genera in ileum in the NC and HC groups (Table 4). The relative abundances of *Clostridium*_*sensu*_*stricto*_1 (*p* = 0.022), *Eubacterium*_*nodatum*_group (*p* = 0.026), *Ruminococcus*_*gauvreauii*_group (*p* = 0.026), *Eubacterium*_*coprostanoligenes*_group (*p* = 0.034), *Ruminococcus* 1 (*p* = 0.031) and *Ruminococcus*_1 (*p* = 0.080) were greater or tended to be greater in the ileum of the HC group compared to that in the NC group. In contrast, the relative abundance of *Anaerotruncus* was lower (*p* = 0.012) in the ileum of the HC group than that in the NC group. Additionally, the relative abundance of *Senegalimassilia* and *Peptoclostridium* tended to be lower in the HC group (*p* < 0.10) than in the NC group.

### 3.3. Function Prediction of Ileal Microbiota Using Picrust

**Figure 3:**
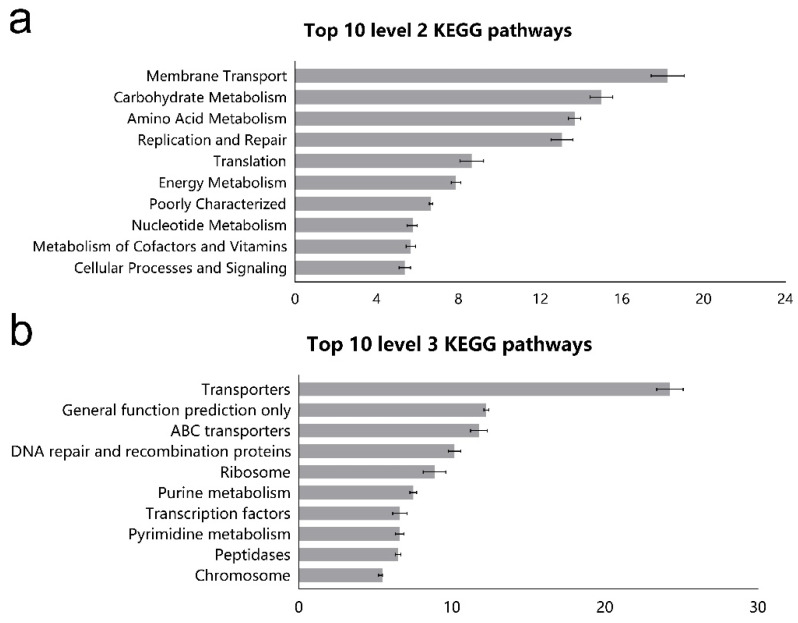
Top 10 predicted metagenomic functions at (**a**) level 2 and (**b**) level 3 of the KEGG (Kyoto Encyclopedia of Genes and Genomes) pathways in ileal bacteria of goats fed the normal and high concentrate diets. The bars stand for the percentage of relative abundance of each predicted function.

Functional prediction of ileal microbiota was analyzed by using 16S rRNA marker gene sequences through PICRUSt (Phylogenetic Investigation of Communities by Reconstruction of Unobserved States) against KEGG (Kyoto Encyclopedia of Genes and Genomes) pathways. As shown in Figure 3a, within the top 10 KEGG pathways, membrane transport pathway was associated with environmental information processing. Five other pathways, including the metabolism of carbohydrates, amino acids, energy, nucleotides, cofactors and vitamins were associated with nutrients metabolism. The remaining three pathways, including replication and repair, translation, and cellular processes and signaling, were associated with genetic information processing. In total, 149 individual pathways were predicted (abundance of >0.1%), and the top 10 most-abundant pathways consisted of three pathways related to environmental information processing (as shown in Figure 3b), including transporters ATP (adenosine triphosphate)-binding cassette (ABC) transporters and transcription factors; four pathways related to genetic information processes, including general function prediction only, DNA repair and recombination proteins, ribosome and chromosome; and three pathways related to metabolism, including purine metabolism, pyrimidine metabolism, and peptidases.

A comparison of different pathways (Table 5) revealed that the HC diet influenced the functional potentials of ileal digesta microbiota. Specifically, compared with the NC group, the HC diet greatly improved pentose and glucuronate interconversions, pentose phosphate pathway, nicotinate and nicotinamide metabolism and biosynthesis of ansamycins (*p* < 0.01). In contrast, the HC diet remarkably decreased abundance of four pathways associated with novobiocin biosynthesis, tropane, piperidine and pyridine alkaloid biosynthesis, bacterial chemotaxis and nitrotoluene degradation (*p* < 0.01). Besides, the HC diet notably increased histidine metabolism, pyruvate metabolism, starch and sucrose metabolism, other glycan degradation, glycerolipid metabolism, vitamin b6 metabolism, tetracycline biosynthesis, polycyclic aromatic hydrocarbon degradation and significantly decreased valine, leucine and isoleucine degradation, butanoate metabolism, flagellar assembly, riboflavin metabolism, beta-alanine metabolism, two-component system (*p* < 0.05). Lastly, the HC group had a tendency to increase valine, leucine and isoleucine biosynthesis, galactose metabolism, fatty acid biosynthesis and tended to decrease cysteine and methionine metabolism compared to those in the NC group (*p* < 0.10).

### 3.4. Metabolites and Biochemical Parameters in the Ileal Digesta

From Table S1 we can infer that there were no differences in the ratio of acetate and propionate, acetate, propionate and butyrate concentration of ileal digesta in two groups (*p* > 0.05). The pH in ileal content decreased significantly when dietary concentrate ratio increased from 55% to 90% (7.11 ± 0.07 vs. 6.63 ± 0.06; *p* = 0.004), and concentration of total VFA was significantly increased in ileal digesta (3.67 ± 1.18 vs. 7.44 ± 2.93 mM; *p* = 0.030). As shown in Table 6, compared with the NC group, the HC group contained higher ALP concentrations in the ileal digesta (5.95 ± 2.26 vs. 8.76 ± 0.83 U/mL; *p* = 0.017), and LDH were not detected in the intestinal digesta of HC-fed goats (4.83 ± 2.64 vs. 0 U/L; *p* = 0.006). There were no differences on LPS, AST, ALT activities and LACT concentrations between the NC and HC group (*p* > 0.05). Compared to those fed the NC diet, HC-fed goats had higher AMY activity in ileal digesta (195 ± 18 vs. 272 ± 53 U/L; *p* = 0.014).

### 3.5. Relationship among the Bacterial Community and Metabolites and Biochemical Indices

**Figure 4:**
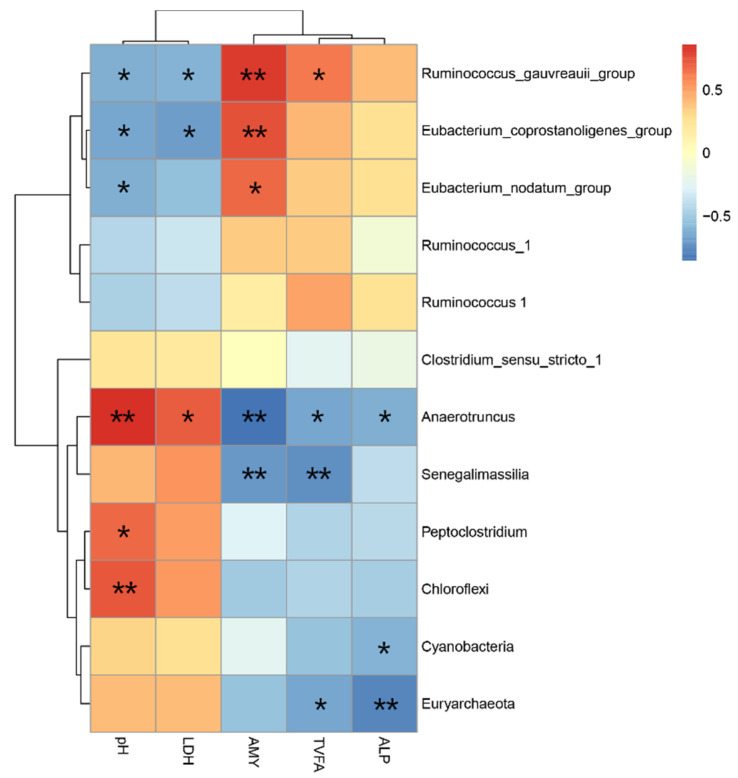
Correlation analysis among the ileal pH, LDH (lactate dehydrogenase), AMY (amylase), TVFA (total volatile fatty acids) and ALP (alkaline phosphatase) concentration and twelve associated species bacteria of difference at the level of genus are shown. Cells are colored based on Pearson’s correlation coefficient between the metabolites and associated bacteria of difference in the intestine (red indicates negative correlation, and blue indicates positive correlation, * and ** respectively indicate *p* < 0.05 and *p* < 0.01).

A correlation analysis was carried out to determine whether there were any relationships among intestinal flora and pH, LDH, AMY, TVFA, and ALP in the ileum. The abundance of the bacterial community at the genus level and variables of metabolites were regarded as correlated with each other if their correlation coefficients were above 0.55. As shown in Figure 4, the abundance of *Ruminococcus_gauvreauii*_group (*r* = −0.615, *p* = 0.033), *Eubacterium_coprostanoligenes*_group (*r* = −0.664, *p* = 0.018) and *Eubacterium_nodatum*_group (*r* = −0.615, *p* = 0.033) was negatively correlated with the pH value. However, *Peptoclostridium* (*r* = 0.685, *p* = 0.014), *Anaerotruncus* (*r* = 0.860, *p* < 0.01) and *Chloroflexi* (*r* = 0.734, *p* < 0.01) were positively correlated with the pH in the ileum. The concentration of LDH was positively correlated with the *Anaerotruncus* (*r* = 0.707, *p* = 0.010) and negatively correlated with the abundance of *Eubacterium_coprostanoligenes*_group (*r* = −0.684, *p* = 0.014) and *Ruminococcus_gauvreauii*_group (*r* = −0.610, *p* = 0.035) in the ileum. In addition, the abundance of three genera, including *Eubacterium_coprostanoligenes*_group (*r* = 0.748, *p* = 0.005), *Eubacterium_nodatum*_group (*r* = 0.685, *p* = 0.014), *Ruminococcus_gauvreauii*_group (*r* = 0.818, *p* < 0.01) was positively correlated with the activity of AMY. Nevertheless, *Anaerotruncus* (*r* = −0.853, *p* < 0.01) and *Senegalimassilia* (*r* = −0.713, *p* < 0.01) abundances were negatively correlated with the AMY activity in the ileum. The abundance of *Ruminococcus_gauvreauii*_group (*r* = 0.622, *p* = 0.031) was positively associated with the TVFA; the abundance of three taxa (*Anaerotruncus* (*r* = −0.650, *p* = 0.022), *Senegalimassilia* (*r* = −0.734, *p* < 0.01) and Euryarchaeota (*r* = −0.650, *p* = 0.022) was negatively correlated with the TVFA concentration in the ileum. There were only three genera (Anaerotruncus (*r* = −0.629, *p* = 0.028), Cyanobacteria (*r* = −0.601, *p* = 0.039) and Euryarchaeota (*r* = −0.769, *p* < 0.01) significantly correlated with ALP activity.

### 3.6. Intestinal Morphology

**Figure 5:**
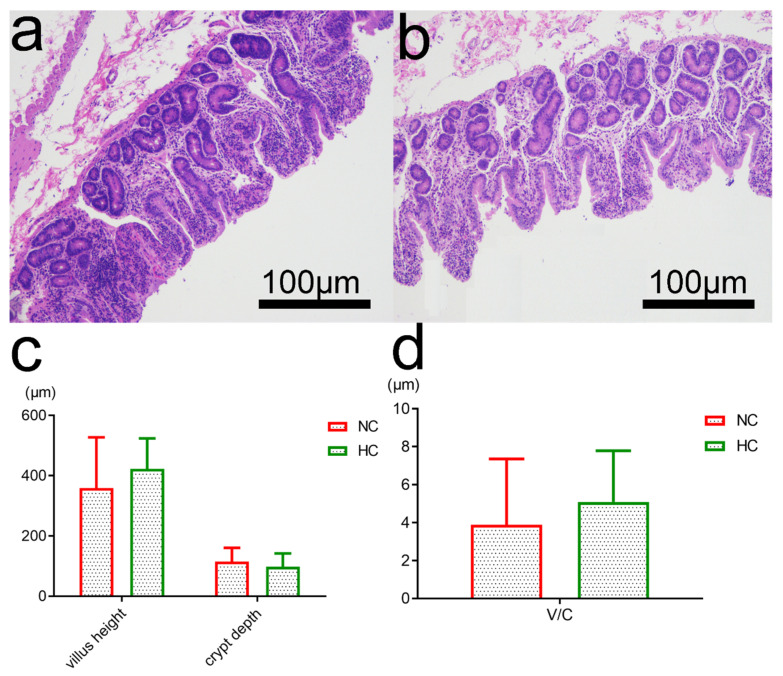
Intestinal morphology of the goats fed normal-concentrate (NC) and high-concentrate (HC) diets. Light microscopy cross-section of ileal tissues from a representative NC-fed goat (**a**) and HC-fed goat (**b**) and the scale bar = 100 μm. Statistical analyses of villlus height and crypt depth (**c**), (*p* > 0.1) and the ratio of villus height to crypt depth of ileal morphology in goats (**d**), (*p* = 0.530).

Representative light micrographs of the cross sections of ileal morphology of the NC diet-fed and HC diet-fed goats were shown in Figure 5a,b. Ileal morphology of goats in the NC group was more integrated than those in the HC group. In the NC diet-fed goats, the orifices of crypts were circular in outline and the intercryptal surface was partially covered by an irregular layer of mucus, and the microvillus clusters were clear and well organized (Figure 5a). In contrast, the ileum of HC diet-fed goats exhibited sloughing of the epithelial surface and layer of mucus irregularly distributed than that of goats in the NC group (Figure 5b). Although ileal tissue in the NC group showed shorter villus height (V) and deeper crypt depth (C) in Figure 5c, there were no differences on villus height, crypt depth and V/C (Figure 5d) between the NC and HC groups (*p* > 0.05).

## 4. Discussion

The influence of feeding goats with rice on the gut micro-environment and intestinal health is still unknown. In previous studies by our group and others, the HC diet appeared to disrupt the balance of the ruminal microbiota in ruminants by Illumina MiSeq sequencing methods [9,24,25]. However, little information was available on microbial response of the small intestine to the HC diet using a high-throughput method [26]. In the present study, we used high-throughput 16S rRNA gene sequencing to assess the shifts in the microbial adaptation of goat’s ileum in response to a HC diet. Our data provide a detailed picture of the ileal metabolites related to microorganisms in goats.

The intestinal microbial community imparts specific functions in the host such as nutrient absorption, drug metabolism, maintenance of structural integrity of the gut barrier, immunoregulation, and protection against pathogens [27]. In accordance with a previous study which reported that feeding an HC diet could reduce the bacterial richness and functionality of the microbiota in the digestive tract of ruminants [28], the Chao1 and Shannon values in our results showed a sharp decline in the ileum of goats fed an HC diet. In addition, the results of the PCA and Anosim analyses further revealed the difference in bacterial diversity composition between the NC and HC groups, also indicated that feeding a HC diet changed the bacterial diversity in the ileum.

In agreement with earlier studies of the intestinal bacterial communities of goats [17,19], our results revealed that the Firmicutes was the major phylum in the ileal digesta of goats. Bacteria belonging to the Firmicutes are known for a fermentative metabolism and degradation of carbon sources, protein and amino acid [29]. Thus, the high abundance of Firmicutes in ileal digesta bacteria emphasized that Firmicutes played a role in the utilization of carbohydrates, protein and amino acid. Tao et al. [30] deduced that the second-most abundant bacterial community, Bacteroidetes of Gram-negative bacteria were increased in the hindgut by a HC diet. However, in the current research, Tenericutes was the second most abundant bacterial community in the ileum. It is possible that different digestive location or diet composition has different microbial flora, because it is known that the composition of the gut microbial community is related to the diet type or host phylogeny [31]. It was previously found that LPS was produced abundantly in the digesta derived from Bacteroides spp. [32]. In the current study, the Bacteroides population was less than 1%. Maybe that could explain why there was no difference in LPS concentration in the ileal digesta. Jenkins et al. [33] reported that species belonging to the family of Christensenellaceae were common in the rumen and that these species played a key role in preserving gastrointestinal structure and function. Compared with the NC group, we detected no difference in proportion of the Christensenellaceae_R-7_group (Family, butyrate producer) in ileal microbial response of goats to the HC diet. Besides, our results somehow showed no difference in butyrate concentration between the two groups. Generally speaking, Cyanobacteria phylum is not regarded as intestinal bacteria in goats although it had been detected previously in human and other animal gut [34]. In this study, the abundance of the Cyanobacteria phylum had a downward trend. Ley et al. [35] reported that there was a logical intestine-associated branch rooted deep in the Cyanobacteria group, and this group may represent descendants of non-photosynthetic ancestral cyanobacteria that have adapted to life in the gastrointestinal tract of animals.

In the present study, a great number of Ruminococcaceae was found in the ileum digesta of the HC group. This is not consistent with the findings recorded in the cecum of goats fed a high-grain diet [36]. This difference may be due to different responses of ileal microbes to diets, and the HC diet provided suitable nutrient conditions for Ruminococcaceae to grow [37], resulting in its rise. *Clostridium* is well known as an intestinal colonizer. It has been reported that some *Clostridium* spp., as opportunistic pathogens, are causative agents of intestinal enteric diseases in goats [38]. Moreover, *Clostridium* phylotypes can negatively impact the intestinal barrier [39]. An increase in the richness of *Clostridium*_*sensu*_*stricto*_1 in the present study hints that feeding a HC diet may have a harmful effect on the ileal health of goats. Liu et al. [36] found that a HC diet in goats increased the population of Turicibacter, Treponema and Prevotella in the cecum. Turicibacter in the gut may cause subclinical infection or have some other adverse reactions on the gastrointestinal tract [40], however, the present study revealed that there was no difference in Turicibacter, and Treponema and Prevotella were not detected in the ileum of goats. In addition, HC diet contained a greater number of *Ruminococcus*_*gauvreauii*_group, *Ruminococcus* 1, and *Ruminococcus*_1 in the ileal digesta than that of the NC group. *Ruminococcus* is the most dominant genus found in the large intestine of healthy sheep [41] and plays a vital role in degrading starch [42,43]. So, the *Ruminococcus* spp. elevation in the ileum digesta may suggest that HC diet improves the ability of the ileal bacteria to digest starch.

It has been revealed that the gut microbiota makes an essential functional contribution in maintaining the health of the gastrointestinal tract for the host [44,45]. Our results showed that the most abundant gene categories in the ileum of goats were related to the function of membrane transport, amino acid metabolism, and energy metabolism, which is similar to the results of previous studies that were conducted in sheep [23]. The system of membrane transport is essential to communicate with the tissues and environment and import molecules into cells and export waste from the cells [46]. PICRUSt from the current study suggested that the relative abundance of genes associated with amino acid metabolism, carbohydrate metabolism, and lipid metabolism was up-regulated by HC diet, indicating that HC diet enhanced the metabolic function of microbes. This shift of coordination in gene expression implies a selective change in metabolic pathways favoring the use of carbohydrates as fuel to sustain energy expenditure for the ileal microflora. Besides, the ileal microflora was predicted to have greater capabilities for replication and repair, and this may be due to the rapid turnover rate of ileal bacteria. Small-intestinal microbiota are proven to be the key sensor of dietary signals that allow the host to adapt to variations in lipid digestion and absorption, and a reference strain *Clostridium* genus can increase oleic acid uptake and the expression of genes involved in triglyceride synthesis [47]. Thus, the observed increment in the *Clostridium*_*sensu*_*stricto*_1 may be related to regulating lipid metabolism in the ileum of goats. The nicotinate and nicotinamide metabolism and vitamin B6 metabolism are essential to maintain sufficient biological response involved in the metabolism of carbohydrates, lipids and proteins [48]. So, we observed that the enhanced vitamin-related pathway was accompanied by boosting the metabolic activity of major nutrients. In fact, vitamins cannot be synthesized by the animal body but can be synthesized by commensal bacteria [49], and vitamins in the ileum mainly originate from the diet and commensal microorganisms. An increase in *Clostridium* abundance, which represents vitamin producers, also confirmed this viewpoint [50]. In addition, the HC diet altered the ileal microorganism populations in particular carbohydrate and protein degraders such as *Clostridium*_*sensu*_*stricto*_1 and some Ruminococcaceae spp. (*Eubacterium*_*coprostanoligenes*_group, *Ruminococcus* 1 and *Ruminococcus*_1). So ileal microbial response to a HC diet involved upregulation of most genes involved in metabolism of macronutrients (carbohydrates, lipids, and proteins) and micronutrients (vitamins), reflecting an augmentation of the metabolic activity in the ileum.

Previous studies on VFA in the rumen and hindgut of ruminants have explored feeding a HC diet have been explored [16,17], however, little information is available on changes of VFA pattern in the ileum during digestion of a HC diet. Our previous research revealed that the HC diet decreased the pH and increased TVFA concentrations in the ileum [19]. Interestingly, about a two-fold increase in TVFA concentrations was detected in the HC group compared with the NC group. Thus, we inferred that the higher VFA in the ileum of HC-fed goats may be due to rapid degradation of carbohydrates or the excessive VFA escaped from the rumen into the ileum of goats. The over-fermentation of VFA in hindgut increases the osmotic pressure of digesta, and the high osmotic pressure partially damages the intestinal epithelium [16], so the damage may be similar in the ileum. Studies of Steele et al. [51] and Liu et al. [52] have shown that feeding ruminants with a HC diet will increase the concentration of LPS in the rumen and cause ruminal epithelial damage. Besides, the intestinal epithelium is composed of only a single layer of epithelial cells, whereas the rumen epithelium consists of a multilayered squamous epithelium [6]. Thus, differences between the structures of the ileal and ruminal epithelia are likely to make the ileal epithelium more susceptible to damage than the rumen responding to the HC diet. Nonetheless, Zebeli et al. [53] indicated that LPS is released by Gram-negative bacteria after lysis at low pH and there were no significantly negative correlations between the concentration of LPS in the ileum and the proportion of Gram-negative bacteria. These findings demonstrate that an HC diet feeding results in a low ileal pH and that the latter could not lead to the death and lysis of Gram-negative bacteria in the ileum, therefore, it had not increased the concentration of LPS in the ileum. In addition, the elevated level of enzymes (AST, ALT, and ALP) indicated some damage or changes in membrane permeability [54], rather than damage to the intestinal villus and crypt in the HC group. The abundance of the genera Anaerotruncus, Cyanobacteria, and Euryarchaeota was negatively correlated with ALP activity, suggesting that they might be involved in the ALP metabolism of the ileum. However, what role these genera played in the intestinal barrier needs to be clarified. The LDH is released during tissue damage and as a marker of pathological changes. In particular, feedback inhibition by LDH can reduce the conversion of pyruvate to LACT at high lactate concentration [55]. Our data display no significant difference in ileal LACT concentration between treatments and did not detect LDH in the HC diet. Maybe the LDH is all used to neutralize LACT and further investigation is needed to explain this phenomenon. AMY activity of fermenting microorganisms is an important factor in the fermentation of starch to lactate [56]. Previous studies have demonstrated that the concentration of AMY was related to dry matter intake and nutrient composition, and more starch intake may enhance the AMY concentration in the small intestine [57,58]. In this study, we found that the HC diet significantly increased the activity of AMY, and *Eubacterium*_*coprostanoligenes*_group, *Eubacterium*_*nodatum*_group and *Ruminococcus*_*gauvreauii*_group were positively correlated with the activity of AMY in the ileum. These results indicate that more starch may flow into the ileum from the rumen due to the HC diet.

Previous studies have revealed that feeding a HC diet to ruminants caused a high risk of damage to the histological integrity and functions of the ruminal epithelium in ruminants [51,59]. However, there are few reports on the influence of a HC diet on ileal epithelial structure and bacterial function. In this study, after five weeks of feeding, the ileal epithelial structure of HC-fed goats was changed slightly. The HC diet did not significantly alter the villus height and crypt depth of ileum and caused only little sloughing of the epithelial structure. Overall, the findings indicate that, in the small intestine of goats fed with a HC diet, a slightly damaged epithelial barrier function may be associated with changes in VFA fermentation, enzyme metabolism and composition of microbiota. Therefore, these findings indicate that the HC diet may have a potentially adverse impact on ileal function.

## 5. Conclusions

In summary, HC diet reduced the microbial diversity and elevated the abundance of five genera related to carbohydrate and protein degraders and affected several pathways together with utilization of nutrient and interfered with metabolic function in the ileum of goats. Our study provides a more comprehensive understanding of the function in the ileal microbes, including novel linkages among bacterial composition, functional potential, and host metabolic responses occurring in the ileum.

## Figures and Tables

**Table 1:** Ingredients and nutrient levels of the experimental diets for sixteen goats (air-dried basis).

Items	NC ^1^ (*n* = 8)	HC ^2^ (*n* = 8)
Ingredients composition (%)		
Rice straw	45.0	10.0
Rice with shell	33.2	54.3
Soybean meal	9.60	15.7
Wheat bran	6.00	9.80
Fat powder	3.20	5.20
Calcium carbonate	0.500	0.800
Calcium bicarbonate	1.10	1.80
Sodium chloride	0.600	1.00
Premix ^3^	1.00	1.40
Nutrient levels ^4^, % of DM (dry matter)		
Crude protein	13.5	17.6
Crude ash	9.34	9.12
Crude fat	4.18	6.01
Neutral detergent fiber	49.8	38.4
Acid detergent fiber	36.5	9.51

^1^ NC: Normal-concentrate diet; ^2^ HC: High-concentrate diet; ^3^ Premix Composition per kg diet: 68 mg FeSO_4_·H_2_O, 44 mg CuSO_4_·5H_2_O, 411 µg CoCl_2_·6H_2_O, 1.70 mg KIO_3_, 211 mg MnSO_4_·H_2_O, 126 mg ZnSO_4_·H_2_O, 56 µg Na_2_SeO_3_, 462 mg MgSO_4_·7H_2_O, 737 IU vitamin A, 8.29 mg vitamin E, 5.1 g carrier zeolite powder; ^4^ Nutrient levels were measured values and consistent with the methods we mentioned earlier [20].

**Table 2:** The intra-assay variation and inter-assay variation of kits for twelve goats’ metabolites.

Items	Intra-Assay Variation	Inter-Assay Variation
LACT ^1^	cv %: ≤2%	cv %: ≤3%
LDH ^2^	cv %: ≤3%	cv %: ≤5%
ALT ^3^	cv %: ≤3%	cv %: ≤4%
AST ^4^	cv %: ≤3%	cv %: ≤4%
ALP ^5^	cv %: ≤2%	cv %: ≤3%
AMY ^6^	cv %: ≤2%	cv %: ≤3%

^1^ LACT: lactic acid; ^2^ LDH: lactate dehydrogenase; ^3^ ALT: alanine aminotransferase; ^4^ AST: aspartate aminotransferase; ^5^ ALP: alkaline phosphatase; ^6^ AMY: amylase.

**Table 3:** Phylum-level composition (%) of the ileal bacterial community of twelve goats fed normal-concentrate (NC) and high-concentrate (HC) diets.

Phylum	NC ^1^ (*n* = 6)	HC ^2^ (*n* = 6)	*p*-Value
Actinobacteria	2.88 ± 2.21	2.37± 1.06	0.624
Bacteroidetes	0.90 ± 2.08	1.07 ± 0.80	0.853
Chloroflexi	1.08 ± 1.21	0.02 ± 0.02	0.058
Cyanobacteria	0.83 ± 0.66	0.25 ± 0.42	0.090
Elusimicrobia	0.91 ± 0.93	0.96 ± 2.16	0.954
Euryarchaeota	0.64 ± 0.46	0.23 ± 0.10	0.055
Firmicutes	79.5 ± 10.3	80.2 ± 18.6	0.945
Lentisphaerae	1.36 ± 0.92	1.13 ± 1.25	0.721
Proteobacteria	0.83 ± 0.82	1.12 ± 1.23	0.631
Saccharibacteria	2.38 ± 1.64	1.16 ± 1.38	0.191
Tenericutes	5.92 ± 4.20	10.5 ± 8.4	0.570
Verrucomicrobia	2.50 ± 2.00	0.86 ± 0.66	0.220

^1^ NC: Normal-concentrate diet; ^2^ HC: High-concentrate diet.

**Table 4:** Genus-level composition of the ileal bacterial community of twelve goats fed normal-concentrate (NC) and high-concentrate (HC) diets.

Classification Levels of Bacteria			Abundance (%)		*p*-Value
Phylum	Family	Genus	NC ^1^ (*n* = 6)	HC ^2^ (*n* = 6)	
Actinobacteria	Coriobacteriaceae	*Senegalimassilia*	1.39 ± 1.28	0.43 ± 0.21	0.099
Elusimicrobia	Elusimicrobiaceae	*Elusimicrobium*	0.91 ± 0.93	0.96 ± 2.16	0.956
Firmicutes	Christensenellaceae	Christensenellaceae_R-7_group	18.2 ± 12.1	12.3 ± 5.11	0.149
	Clostridiaceae_1	*Clostridium*_*sensu*_*stricto*_1	0.26 ± 0.11	1.13 ± 0.62	0.022
	Erysipelotrichaceae	*Turicibacter*	0.82 ± 0.62	1.19 ± 1.73	0.641
	Family_XIII	*Eubacterium*_*nodatum*_group	0.53 ± 0.28	1.26 ± 0.63	0.026
	Family_XIII	Family_XIII_AD3011_group	2.21 ± 0.99	2.31 ± 0.67	0.846
	Family_XIII	*Mogibacterium*	2.59 ± 2.26	2.72 ± 1.35	0.903
	Lachnospiraceae	*Acetitomaculum*	1.12 ± 0.89	1.57 ± 1.56	0.556
	Lachnospiraceae	*Eubacterium*_*ventriosum*_group	0.23 ± 0.45	1.53 ± 2.35	0.236
	Lachnospiraceae	Lachnospiraceae_NK3A20_group	2.20 ± 1.59	2.37 ± 1.43	0.850
	Lachnospiraceae	*Ruminococcus*_*gauvreauii*_group	0.28 ± 0.28	1.42 ± 0.91	0.026
	Peptostreptococcaceae	*Peptoclostridium*	5.71 ± 4.44	1.40 ± 2.27	0.061
	Peptostreptococcaceae	*Romboutsia*	7.71 ± 4.53	5.00 ± 6.05	0.402
	Ruminococcaceae	*Anaerotruncus*	1.79 ± 0.99	0.23 ± 0.10	0.012
	Ruminococcaceae	*Eubacterium*_*coprostanoligenes*_group	1.07 ± 0.59	4.51 ± 3.38	0.034
	Ruminococcaceae	Ruminococcaceae_NK4A214_group	6.06 ± 3.24	7.79 ± 4.68	0.472
	Ruminococcaceae	Ruminococcaceae_UCG-001	0.20 ± 0.28	1.24 ± 1.92	0.247
	Ruminococcaceae	Ruminococcaceae_UCG-014	3.32 ± 3.41	9.36 ± 9.22	0.180
	Ruminococcaceae	*Ruminococcus* 1	0.18 ± 0.36	1.00 ± 0.72	0.031
	Ruminococcaceae	*Ruminococcus*_1	0.30 ± 0.23	0.99 ± 0.76	0.080
	Ruminococcaceae	*Ruminococcus*_2	1.77 ± 1.62	5.24 ± 4.82	0.144
	Ruminococcaceae	*Saccharofermentans*	9.76 ± 2.15	5.70 ± 7.06	0.495
Saccharibacteria	Unknown	*Candidatus*_*Saccharimonas*	2.38 ± 1.64	1.16 ± 1.38	0.191
Tenericutes	Mycoplasmataceae	Mycoplasma	3.92 ± 4.79	8.65 ± 18.8	0.564
		Unidentified	14.6 ± 6.96	9.32 ± 2.45	0.127

^1^ NC: Normal-concentrate diet; ^2^ HC: High-concentrate diet.

**Table 5:** KEGG pathways that showed different abundances (%) between ileal digesta microbiota in twelve goats fed the normal-concentrate (NC) and high-concentrate (HC) diets.

Level 2	Level 3	Pathway ID	NC ^1^ (*n* = 6)	HC ^2^ (*n* = 6)	*p*-Value
Amino acid metabolism	Cysteine and methionine metabolism	ko00270	1.01 ± 0.03	0.97 ± 0.06	0.055
	Histidine metabolism	ko00340	0.62 ± 0.02	0.65 ± 0.01	0.010
	Valine, leucine and isoleucine biosynthesis	ko00290	0.75 ± 0.02	0.79 ± 0.04	0.050
	Valine, leucine and isoleucine degradation	ko00280	0.22 ± 0.03	0.19 ± 0.02	0.037
Biosynthesis of other secondary metabolites	Novobiocin biosynthesis	ko00401	0.16 ± 0.005	0.14 ± 0.01	0.004
	Tropane, piperidine and pyridine alkaloid biosynthesis	ko00960	0.14 ± 0.005	0.13 ± 0.007	0.004
Carbohydrate metabolism	Butanoate metabolism	ko00650	0.77 ± 0.02	0.70 ± 0.06	0.010
	Galactose metabolism	ko00052	0.63 ± 0.03	0.68 ± 0.05	0.055
	Pentose and glucuronate interconversions	ko00040	0.48 ± 0.03	0.53 ± 0.01	0.004
	Pentose phosphate pathway	ko00030	0.86 ± 0.03	0.95 ± 0.03	0.004
	Pyruvate metabolism	ko00620	1.12 ± 0.03	1.16 ± 0.04	0.037
	Starch and sucrose metabolism	ko00500	0.94 ± 0.03	1.02 ± 0.04	0.010
Cell motility	Bacterial chemotaxis	ko02030	0.70 ± 0.12	0.58 ± 0.02	0.006
	Flagellar assembly	ko02040	0.70 ± 0.11	0.56 ± 0.07	0.037
Glycan biosynthesis and metabolism	Other glycan degradation	ko00511	0.12 ± 0.01	0.14 ± 0.01	0.025
Lipid metabolism	Fatty acid biosynthesis	ko00061	0.52 ± 0.02	0.55 ± 0.03	0.055
	Glycerolipid metabolism	ko00561	0.42 ± 0.02	0.46 ± 0.02	0.016
Metabolism of cofactors and vitamins	Nicotinate and nicotinamide metabolism	ko00760	0.41 ± 0.01	0.45 ± 0.02	0.004
	Riboflavin metabolism	ko00740	0.21 ± 0.02	0.19 ± 0.02	0.025
	Vitamin B6 metabolism	ko00750	0.17 ± 0.01	0.20 ± 0.02	0.025
Metabolism of other amino acids	beta-Alanine metabolism	ko00410	0.16 ± 0.02	0.14 ± 0.01	0.025
Metabolism of terpenoids and polyketides	Biosynthesis of ansamycins	ko01051	0.12 ± 0.003	0.13 ± 0.003	0.004
	Tetracycline biosynthesis	ko00253	0.18 ± 0.009	0.20 ± 0.01	0.010
Signal transduction	Two-component system	ko02020	1.52 ± 0.11	1.37 ± 0.07	0.010
Xenobiotics biodegradation and metabolism	Nitrotoluene degradation	ko00633	0.13 ± 0.004	0.09 ± 0.02	0.004
	Polycyclic aromatic hydrocarbon degradation	ko00624	0.09 ± 0.005	0.11 ± 0.007	0.016

^1^ NC: Normal-concentrate diet; ^2^ HC: High-concentrate diet.

**Table 6:** Effects of high-concentrate diet on ileal parameters in twelve goats.

Items	NC ^1^ (*n* = 6)	HC ^2^ (*n* = 6)	*p* Value
LPS ^3^ (EU/mL)	0.41 ± 0.04	0.36 ± 0.05	0.136
LACT ^4^ (mmol/L)	0.14 ± 0.05	0.20 ± 0.14	0.343
LDH ^5^ (U/L)	4.83 ± 2.64	ND ^6^	0.006
ALT ^7^ (U/L)	2.00 ± 0.58	1.55 ± 0.56	0.206
AST ^8^ (U/L)	7.05 ± 4.86	6.17 ± 4.18	0.743
ALP ^9^ (U/mL)	5.95 ± 2.26	8.76 ± 0.83	0.017
AMY ^10^ (U/L)	195 ± 18	272 ± 53	0.014

^1^ NC: Normal-concentrate diet; ^2^ HC: High-concentrate diet; ^3^ LPS: Lipopolysaccharide; ^4^ LACT: Lactic acid; ^5^ LDH: Lactate dehydrogenase; ^6^ ND, not detectable; ^7^ ALT: Alanine aminotransferase; ^8^ AST: Aspartate aminotransferase; ^9^ ALP: Alkaline phosphatase; ^10^ AMY: Amylase.

## Supplementary Materials

The following are available online at https://www.mdpi.com/2076-2615/10/8/1261/s1, Table S1: Effects of high-concentrate diet on the ileal pH, molar proportions of VFA in goats, Table S2: Family-level composition (%) of the ileal bacterial community in the NC and HC goats.
