# Supplementary material for: Diet with a High Proportion of Rice Alters Profiles and Potential Function of Digesta-Associated Microbiota in the Ileum of Goats
Source: Animals (Basel). 2020 Jul 24;10(8):1261. doi: 10.3390/ani10081261 (PMC7460243; doi:10.3390/ani10081261)
Supplement: Supplementary file 1 [file animals-10-01261-s001.pdf]

Table S1. Effects of high concentrate diet on the ileal pH, molar proportions of VFA in goats[1]

Items	NC[2]	HC[3]	*P* value
pH	7.11 ± 0.07	6.63 ± 0.06	0.004
TVFA[4] (mM)	3.67 ± 1.18	7.44 ± 2.93	0.030
Acetate: Propionate	39.2 ± 9.2	28.0 ± 8.5	NS[5]
Individual VFA molar percentage (%)			
Acetate	95.3 ± 1.4	94.2 ± 3.0	NS
Propionate	2.57 ± 0.77	3.68 ± 1.33	NS
Butyrate	2.15 ± 0.62	2.16 ± 1.89	NS

[1] the data from our previous work (DOI: 10.1021/acs.jafc.8b05591); [2]NC: normal concentrate diet; [3]HC: high concentrate diet; [4]TVFA, total volatile fatty acids; [5]NS, not significant.

Table S2. Family-level composition (%) of the ileal bacterial community fed normal and high concentrate goats

Family	NC[1]	HC[2]	*P* value
Ruminococcaceae	27.5 ± 8.11	37.6 ± 11.7	NS[3]
Christensenellace	18.9 ± 12.5	12.4 ± 5.17	NS
Lachnospiraceae	9.78 ± 4.65	11.5 ± 3.91	NS
Peptostreptococca	13.4 ± 7.56	6.40 ± 8.11	NS
Family_XIII	6.29 ± 3.06	7.99 ± 2.09	NS
Mycoplasmatacea	4.06 ± 5.01	8.74 ± 19.0	NS
unidentified	4.86 ± 3.01	2.76 ± 1.58	NS
Erysipelotrichace	1.90 ± 0.90	2.31 ± 1.80	NS
Coriobacteriaceae	2.29 ± 1.79	1.80 ± 0.98	NS
Unknown_Family	2.38 ± 1.64	1.16 ± 1.38	NS
vadinBE97	1.17 ± 0.96	0.97 ± 1.17	NS
Elusimicrobiaceae	0.91 ± 0.93	0.96 ± 2.16	NS
Clostridiaceae_1	0.47 ± 0.30	1.23 ± 2.39	NS
Anaerolineaceae	1.08 ± 1.21	0.02 ± 0.02	0.058

[1]NC: normal concentrate diet; [2]HC: high concentrate diet; [3]NS, not significant ($p > 0.10$).
